# Effects of Chinese Dietary Pattern of Fat Content, n-6/n-3 Polyunsaturated Fatty Acid Ratio, and Cholesterol Content on Lipid Profile in Rats

**DOI:** 10.1155/2018/4398086

**Published:** 2018-03-18

**Authors:** Xian-Guo Zou, Yu-Hua Huang, Hong-Yan Li, Tong-Cheng Xu, Ya-Wei Fan, Jing Li, Ze-Yuan Deng

**Affiliations:** ^1^State Key Lab of Food Science & Technology, Nanchang University, Nanchang, Jiangxi 330047, China; ^2^Institute of Agro-Food Science & Technology, Shandong Academy of Agricultural Sciences, Jinan 250100, China; ^3^College of Food Science & Technology, Nanchang University, Nanchang, Jiangxi 330047, China

## Abstract

This study aims to investigate the effect of Chinese diet pattern of fat content (30% or 36.06%), n-6/n-3 polyunsaturated fatty acid (PUFA) ratio (5 : 1 or 9 : 1), and cholesterol content (0.04 or 0.057 g/kg total diet) on lipid profile using a rat model. Results showed that rats' body weights (BWs) were controlled by the simultaneous intakes of cholesterol level of 0.04 g/kg total diet and n-6/n-3 ratio of 5 : 1. In addition, under high-fat diet, increased cholesterol feeding led to increased total cholesterol (TC) and low density lipoprotein cholesterol (LDL-C) levels and decreased triacylglycerols (TG) in rats' plasma. However, high density lipoprotein cholesterol (HDL-C) level and the ratios of HDL-C/LDL-C and HDL-C/TC in rats' plasma increased in response to simultaneous intakes of low n-6/n-3 ratio (5 : 1) and cholesterol (0.04 g/kg total diet) even under high-fat diet. Moreover, as the n-6/n-3 PUFA ratio in the diet decreased, the proportion of n-3 PUFAs increased in plasma, liver, and muscle and resulted in the decrease of n-6/n-3 PUFA ratio.

## 1. Introduction

The amount and type of dietary fats largely influence plasma lipid content and play an important role in diseases prevention and development [1, 2]. In recent decade years, the number of obese and/or overweight people in China was up to 2,600 million and the data are increasing, which was largely associated with the unhealthy diet of high fat and cholesterol [3]. Data from National Nutrition and Health Survey in China showed that the average intake of fat was 76.2 g/60 kg·body weight (BW)·d in the whole country (30% total calories) and 85.6 g/60 kg·BW·d in the urban areas (36.06% total calories) [4]. And the consumption ratio of n-6/n-3 was 9 : 1 in both the whole country and the urban areas [5]. Moreover, the average cholesterol consumption in the whole country and the urban areas was 0.04 and 0.057 g/kg total diet, respectively [4, 5]. It is obvious that the Chinese dietary pattern of fat and cholesterol has surpassed the recommended level and the ratio of n-6/n-3 almost reached the upper level of FAO/WHO standards [6]. Besides, it was found that the increased Chinese dietary pattern of fat and cholesterol was positively associated with the increased morbidity and mortality of cardiovascular disease, with *R*^2^ value of 0.98 and 1.00, respectively [5]. However, to the best of our knowledge, little has been reported regarding the influence of Chinese dietary pattern of fat and cholesterol on lipid metabolism.

Currently, there are many studies, using rat models, investigating the effects of different fatty acid ratios with or without cholesterol treatment on rats' lipid metabolism. For example, Parrish et al. [7] found that there were attenuated perirenal and epididymal fat accumulation and reduction in adipocyte size in rats fed with a high-fat diet consisting of fish oil, compared to those fed with lard. Wong et al. [8] and Balasubramaniam et al. [9] reported that, in rats, dietary long-chain n-3 polyunsaturated fatty acid (PUFA) could reduce both triacylglycerols (TG) and cholesterol plasma concentrations. Yang et al. [10] found that comparing with rat group fed with n-6/n-3 ratio of 20 : 1, rat groups with the ratios of 1 : 1 and 5 : 1 had significantly decreased serum levels of total cholesterol (TC), low density lipoprotein cholesterol (LDL-C), and proinflammatory cytokines. Besides, Huang et al. [11] reported that the cholesterol-added diet (1%) did not impair the response to long-chain n-3 PUFA but increased the plasma cholesterol content and the liver weight. Moreover, Lu and Wu [12] found that added 1% cholesterol to diets led to a reduction of plasma TG and high density lipoprotein (HDL) levels and an increase of liver TG and cholesterol levels.

Therefore, the aim of this study was to investigate the influence of different fat intake, n-6/n-3 ratio, and cholesterol on lipids metabolism using a rat model according to the Chinese diet pattern. BWs, plasma lipid levels, and fatty acid compositions in rats' plasma and tissues were determined and analyzed.

## 2. Methods and Materials

### 2.1. Materials and Chemicals

A total of 72 young male Sprague-Dawley rats (two or three months, 270 ± 33 g at the beginning of the experiment) were purchased from Experimental Animal Center of Jiangxi Medical College, China (approval number: 02196-02). They were randomly divided into 6 groups with 12 rats per group (Table 1) according to the different consumption of fat energy (30 or 36.06%), n-6/n-3 ratio (5 : 1 or 9 : 1), and cholesterol (0.04 or 0.057 g/kg total diet) and then were housed in the individual cages at a constant temperature of 22 ± 1°C with a normal 12 : 12 hours of light dark cycles. The six groups were Experimental Groups 1, 2, 3, and 4 (EG 1, 2, 3, and 4) and Control Groups 1 and 2 (CG 1 and 2). Except for different contents of palm oil and cholesterol in different groups, the compositions of other nutrients in the diet given to rats were identical (Table 2). Besides, rats in each group was intragastric gavage administered with blended oil of 0.8 mL/100 g·BW·d (EG 1, 2, 3, and 4) or 0.6 mL/100 g·BW·d (CG 1 and 2), so as to maintain the dietary consumption of fat energy and n-6/n-3 ratio in different group. The physical blended oil administered in each group consists of different proportions of camellia oil, sunflower oil, and linseed oil (fatty acid compositions shown in Table 3), and their formula in each group was shown in Table 4. All procedures involving animals were approved by the Ethics Committee, Nanchang University, and conducted in accordance with the guidelines of China for the care and use of laboratory animals.

### 2.2. Animal Treatment and Sampling

Before treatment, blood samples (about 0.5–1.0 mL) were collected from rats' tail vein to measure the biochemical indices, such as TC, TG, LDL-C, and HDL-C. TC and TG were measured enzymatically on the automatic biochemistry analyzer (Beckman, CX4) because of their methodology, calibrators, and controls [13]. The very low density lipoprotein cholesterol (VLDL-C) and LDL-C were precipitated using dextran sulfate and magnesium sulfate before HDL-C was measured enzymatically on the same instrument. LDL-C was calculated from these values (TC, HDL-C, and TG) using the formula of Friedewald et al. [14] when the TG level was <4.52 mmol/L.

After 7-day basal chow, there was no difference in BWs or serum lipid profile among the six groups. Then, each group was maintained on the experimental diet for 60 days. Rats had free access to water. BWs were monitored weekly. After 60 days' treatment, rats were fasted overnight (about 12 h, with water ad libitum) to reduce the feeding impact and then sacrificed under diethyl ether anesthesia. Blood samples were collected by cardiac puncture into clean test tubes containing 1% heparin sodium solution (w/w), centrifuged (4°C, 2500 r/min for 15 min) to get the plasma fraction, and then promptly stored at −80°C until analysis. The main organs and tissues (brain, heart, liver, and muscle) were removed and immediately rinsed in phosphate-buffered saline twice and then stored at −80°C until analysis.

### 2.3. Measurement of Biochemical Indices

Plasma lipid levels of TG, TC, LDL-C, and HDL-C after treatment were determined by automatic biochemistry analyzer (CX4, Beckman) as described above.

### 2.4. Fatty Acid Composition Analysis in Blood and Tissues

Total lipids were extracted with chloroform/methanol (v/v, 1 : 1) from the liver, brain, heart, muscle, and plasma according to the method of Folch et al. [15]. The obtained lipids were converted to fatty acid methyl esters (FAMEs) following the method of Lei et al. [16] and then analyzed by gas chromatography (model 6890N, Agilent Technologies, USA) equipped with an autoinjector, a fused-silica capillary column (CP-Sil 88, 100 m × 0.25 mm × 0.2 *μ*m i.d.), and a flame ionization detector (FID) and operated on in splitless mode. The injector and detector temperatures were maintained at 250 and 260°C, respectively. The column temperature was held at 45°C for 3 min and then programmed to 175°C for 27 min at the rate of 13°C/min. The temperature was then further increased to 215°C at the rate of 4°C/min and held for 35 min. The oven temperature program was sustained for 86 min in total. The carrier gas was nitrogen, and the total gas flow rate was 52 mL/min. The injecting sample volume was 1 *μ*L. Analysis of all peaks was accomplished by comparison of their retention times with FAME standards.

### 2.5. Statistical Analysis

Data were presented as mean ± SD and carried out using the Kruskal-Wallis one-way analysis by Statistical Package for the Social Sciences (SPSS) to compare differences between groups. *P* values less than 0.05 (*P* < 0.05) were considered significant.

## 3. Results

### 3.1. BWs

Under different diet of fat, n-6/n-3 ratio, and cholesterol, the change of BWs among six groups showed difference from the fourth week (Table 5). Compared with CG2 fed with fat energy of 30%, average BW in EG2 fed with higher fat energy of 36.06% was significantly increased (*P* < 0.05). Besides, with the same fat energy of 36.06% and n-6/n-3 ratio of 9 : 1, but different cholesterol level of 0.04 or 0.057 g/kg total diet, there was no significant difference (*P* > 0.05) of BWs in EG1 and EG2. Also, with the same fat energy of 30% and n-6/n-3 of 9 : 1, but different cholesterol level of 0.04 or 0.057 g/kg total diet, there was no significant difference (*P* > 0.05) of BWs in CG1 and CG2. However, with the same fat energy of 36.06% and n-6/n-3 of 5 : 1, the average BW in EG3 feeding cholesterol of 0.04 g/kg total diet was significantly lower than that in EG4 feeding cholesterol of 0.057 g/kg total diet (*P* < 0.05). Rats in EG4 fed with low n-6/n-3 ratio (5 : 1) but high cholesterol (0.057 g/kg total diet) showed no significant difference in BWs compared to those in EG1 feeding high n-6/n-3 ratio (9 : 1) but low cholesterol (0.04 g/kg total diet). Interestingly, rats in EG3 taking low n-6/n-3 ratio (5 : 1) and cholesterol (0.04 g/kg total diet) showed significantly decreased BWs compared to those in EG2 taking high n-6/n-3 ratio (9 : 1) and cholesterol (0.057 g/kg total diet). All these results suggested that rats' BW increased as the increase of fat energy, whereas the increase slowed down by reducing the intake of cholesterol under low intake of n-6/n-3 ratio.

### 3.2. The Plasma Lipid Levels

As shown in Table 6, for the groups with the same contents of fat energy (36.06%) and n-6/n-3 (9 : 1), rats in EG2 fed with cholesterol level of 0.057 g/kg total diet showed significantly (*P* < 0.05) higher levels of TC (1.73 mmol/L) in plasma than those in EG1 fed with cholesterol level of 0.04 g/kg total diet (1.28 mmol/L). Likewise, with the same contents of fat energy (36.06%) and n-6/n-3 (5 : 1), the TC content in EG4 (cholesterol of 0.057 g/kg total diet) was 2.06 ± 0.24 mmol/L, which was significantly higher (*P* < 0.05) than that in EG3 (cholesterol of 0.04 g/kg total diet), 1.28 ± 0.21 mmol/L. Besides, significant difference (*P* < 0.05) was found between CG2 (cholesterol of 0.057 g/kg total diet) and CG1 (cholesterol of 0.04 g/kg total diet), containing 1.58 and 1.23 mmol/L of TC, respectively. Moreover, rats in EG3 taking low n-6/n-3 ratio (5 : 1) and cholesterol (0.04 g/kg total diet) showed significantly decreased TC compared to those in EG2 taking high n-6/n-3 ratio (9 : 1) and cholesterol (0.057 g/kg total diet). From these results, it is clear that no matter the fat content in the diet, the higher cholesterol feeding might cause the higher plasma TC in rats. However, TC content in EG3 fed with 5 : 1 n-6/n-3 was similar to that in EG1 fed with 9 : 1 n-6/n-3, suggesting that n-6/n-3 ratio of 5 : 1 could not reduce plasma TC levels.

The TG contents in EG1, EG2, EG3, and EG4 were 0.43, 0.35, 0.46, and 0.38 mmol/L, respectively. The data showed that, with the same fat energy (36.06%) for EG2/EG4 and EG3, TG contents in EG2/EG4 fed with cholesterol of 0.057 g/kg total diet were significantly lower (*P* < 0.05) than that in EG3 fed with cholesterol of 0.04 g/kg total diet. However, TG contents in CG1 (cholesterol of 0.04 g/kg total diet) and CG2 (cholesterol of 0.057 g/kg total diet), with the same fat energy of 30%, were 0.45 and 0.44 mmol/L, respectively, showing no significant difference (*P* > 0.05). There is a possibility that increased intake of cholesterol under high-fat diet could reduce plasma TG.

The contents of LDL-C in EG1, EG2, EG3, EG4, CG1, and CG2 were 0.39, 0.42, 0.28, 0.67, 0.36, and 0.39 mmol/L, respectively. The level of LDL-C in EG4 (cholesterol of 0.057 g/kg total diet) was significantly higher (*P* < 0.05) compared to that in EG3/EG1 (cholesterol of 0.04 g/kg total diet). It can be inferred that the increasing cholesterol content in diet could increase level of LDL-C in plasma.

With the same n-6/n-3 (9 : 1) in EG1/EG2, the content of HDL-C in both was 0.61 mmol/L, showing no significant differences (*P* > 0.05). Similarly, the contents of HDL-C in CG1 and CG2 (9 : 1 n-6/n-3) were 0.54 and 0.50 mmol/L, respectively, showing no significant differences (*P* > 0.05). However, it was 0.63 and 0.99 mmol/L in EG3 and EG4 (5 : 1 n-6/n-3), respectively, which were higher than those in EG1, EG2, CG1, and CG2 (9 : 1 n-6/n-3). Results indicated that the HDL-C level increased with the increase of n-3 PUFAs in the diet.

Furthermore, HDL-C/TC were decreased significantly (*P* < 0.05) in all groups after treatments. Besides, except for EG3, the ratios of HDL-C/LDL-C were also reduced significantly (*P* < 0.05) in all groups after treatments. With n-6/n-3 ratio of 9 : 1, fat energy of 36.06% in EG1 and EG2, or 30% in CG1 and CG2, there was no significant difference in HDL-C/LDL-C. However, with the same fat energy of 36.06% and n-6/n-3 ratio of 5 : 1, the rats in EG3 fed with cholesterol of 0.04 g/kg total diet showed significantly higher ratio of HDL-C/LDL-C (2.24 ± 0.26) than EG4 fed with cholesterol of 0.057 g/kg total diet (1.53 ± 0.26). Besides, under high-fat diet (36.06%), the ratios in EG4 (1.53 ± 0.26) were relatively higher than in EG2 (1.47 ± 0.29), but significantly higher in EG3 (2.24 ± 0.26) than in EG1 (1.52 ± 0.26), respectively, at the same level of cholesterol, difference in n-6/n-3 with 9 or 5. Moreover, rats in EG3 taking low n-6/n-3 ratio (5 : 1) and cholesterol (0.04 g/kg total diet) showed significantly increased HDL-C/LDL-C and HDL-C/TC compared to those in EG2 taking high n-6/n-3 ratio (9 : 1) and cholesterol (0.057 g/kg total diet). These results demonstrated that decreased ratio of n-6/n-3 in diet could increase the ratio of HDL-C/LDL-C in plasma of rats under high-fat diet, and the increase level was significant accompanying low cholesterol feeding.

As shown in Table 7, negative correlations were found between HDL-C/LDL-C and TC (−0.423, 0.000), between HDL-C/LDL-C and LDL-C (−0.634, 0.000), between HDL-C/LDL-C and TG (−0.539, 0.010), and between TC and LDL-C (−0.253, 0.013). Besides, a negative correlation (−0.679, 0.031) was detected between HDL-C and BWs only in EG3 (not shown in the table). However, there was a positive correlation between TC and TG (0.210, 0.042).

### 3.3. Effects of Dietary Fat and Cholesterol on FA Compositions in Plasma

The FAs profile in the diet could affect the FA compositions in rats' plasma (Table 8). When the n-6/n-3 PUFA ratio in the diet decreased (EG 3 and 4), the proportion of n-3 PUFA in plasma increased, thus resulting in the decrease of n-6/n-3 PUFA ratio. Besides, rats fed with n-6/n-3 ratio of 9 : 1 (EG 1 and 2; CG 1 and 2) had significantly higher level of arachidonic acid (ARA) in the plasma compared to that in rats fed with n-6/n-3 ratio of 5 : 1 (EG 3 and 4). Moreover, the proportions of eicosapentaenoic acid (EPA) and docosahexaenoic acid (DHA) in rats feeding n-6/n-3 ratio of 5 : 1 (EG 3 and 4) were higher than those in rats feeding n-6/n-3 ratio of 9 : 1 (EG 1 and 2; CG 1 and 2). Cholesterol in diet can also affect the FA compositions in plasma. Rats fed with higher cholesterol (0.057 g/kg total diet) increased the TMUFAs (mainly oleic acid), but decreased the TSFAs (mainly stearic acid) compared to those in rats feeding cholesterol of 0.04 g/kg total diet. Further, rats in EG3 taking low n-6/n-3 ratio (5 : 1) and cholesterol (0.04 g/kg total diet) showed higher EPA and DHA and lower ARA compared to those in EG2 taking high n-6/n-3 ratio (9 : 1) and cholesterol (0.057 g/kg total diet).

### 3.4. Effects of Dietary Fat and Cholesterol on FA Compositions in Liver and Muscle

The FA compositions in rats' liver and muscle are shown in Table 9. In liver, rats fed with lower n-6/n-3 ratio of 5 : 1 (EG 3 and 4) had significantly higher level of n-3 PUFA (mainly of ALA) and lower ratio of n-6/n-3 compared to rats fed with n-6/n-3 ratio of 9 : 1 (EG 1 and 2; CG 1 and 2). Similarly, the changes of n-3 PUFA and n-6/n-3 ratio in muscle showed the same tendency with liver. Interestingly, the concentrations of *α*-linolenic acid (ALA) in liver and muscle were higher than those in plasma, brain, and heart tissues. However, traces of EPA and DHA were registered in muscle compared to those in other organs.

### 3.5. Effects of Dietary Fat and Cholesterol on FA Compositions in Brain and Heart

The FAs compositions in rats' brain and heart are presented in Table 10. Brain, a unique organ, can accumulate high concentration of DHA because this fatty acid plays a vital role in development and function of nervous system. In this study, ALA in brain was significantly metabolized to DHA as evidenced by the DHA levels in six groups varied from 11.49% to 13.66%. Interestingly, n-6/n-3 ratios in six groups were approximately with value of 1 : 1. Rats in groups fed with different cholesterol of 0.057 or 0.04 g/kg total diet showed no obvious change of FA compositions. Also, rats in EG3 taking low n-6/n-3 ratio (5 : 1) and cholesterol (0.04 g/kg total diet) showed no significant differences in EPA and DHA and n-6/n-3 ratio compared to those in EG2 taking high n-6/n-3 ratio (9 : 1) and cholesterol (0.057 g/kg total diet).

Heart also showed high DHA levels in six groups changing from 12.18% to 13.25%. Besides, the ARA level in heart changed from 15.33% to 18.08%, higher than those in plasma, liver, brain, and muscle. However, traces of ALA were registered in heart. Similar to the results in plasma, rats fed with n-6/n-3 ratio of 9 : 1 (EG 1 and 2; CG 1 and 2) had higher level of ARA in the heart compared to that in rats fed with n-6/n-3 ratio of 5 : 1 (EG 3 and 4). And the higher cholesterol (0.057 g/kg total diet) in diet also increased the total monounsaturated fatty acid (mainly oleic acid) but decreased the total saturated fatty acids (mainly stearic acid) in heart compared to those in rats feeding cholesterol of 0.04 g/kg total diet.

## 4. Discussion

Dietary modification has led to an imbalance in the n-6/n-3 PUFA ratio with an increase in n-6 FAs but a marked reduction in n-3 FAs in diets, resulting in a high n-6/n-3 PUFA ratio [17]. The n-6/n-3 PUFA ratio of 9 : 1 in today's typical Chinese diets is quite different from the n-6/n-3 PUFA ratio of 1 : 1, which was found in human diets many years ago [18]. Therefore, there is necessity to investigate the effects of Chinese dietary pattern of fat content, n-6/n-3 ratio, and cholesterol content on lipid profile and FA compositions in plasma and tissues using a rat model.

Results showed that high-fat diet contributed to the increasing BWs of rats. However, rats fed with high fat, modulating by cholesterol level of 0.04 g/kg total diet and n-6/n-3 ratio of 5 : 1, showed significant decrease in BWs compared to those of rat fed with cholesterol of 0.057 g/kg total diet and n-6/n-3 ratio of 9 : 1, which indicated that, even under high-fat diet, the increase of rat's BWs could be controlled by simultaneously keeping cholesterol and n-6/n-3 at low levels. Supporting our results, Buckley and Howe [19] reported that a dietary intake of n-3 PUFAs may reduce BWs in obese rats and body fat accumulation, specifically visceral fat of rats fed with a high-fat diet. Guelzim et al. [20] showed that n-3 PUFAs could improve the body composition and insulin sensitivity and resulted in rats' weight loss. Haimeur et al. [21] also found that rats fed with high-fat diet had higher adiposity and adipose tissue/BW ratio compared to those in the control group, but these values were reduced by a dietary supplementation of n-3 PUFA.

The plasma levels of TC, TG, HDL-C, and LDL-C in rats varied with diet. The higher cholesterol fed under high-fat diet, the higher TC and LDL-C levels, and the lower TG in rats' plasma. However, under high-fat diet, the HDL-C level and the ratios of HDL-C/LDL-C and HDL-C/TC in rats' plasma increased in response to simultaneous intake of low n-6/n-3 (5 : 1) and cholesterol (0.04 g/kg total diet) compared to those of rats fed with high cholesterol of 0.057 g/kg total diet and n-6/n-3 ratio of 9 : 1. The result was in concurrence with an earlier report that cholesterol feeding in rats could significantly increase the plasma cholesterol levels, whereas it could reduce the levels of plasma TG and phospholipids [11]. Moreover, in our study, we found that, under high-fat diet, the decreased ratio of n-6/n-3 (5 : 1) in diet could not reduce plasma TC levels, which was different from the results of Jeffery et al. [22] reporting a decreased serum cholesterol concentrations as the n-6/n-3 PUFA ratio in the diet decreased. It has been reported that the incidence of atherosclerosis [23] and cardiovascular disease [24] was in close correlation with the high levels of TC, TG, and LDL-C. On the contrary, a high level of HDL-C was cardioprotective [25]. Moreover, among the possible mechanisms that may contribute to the cardiovascular benefits, n-3 PUFAs other than n-6 PUFAs were found to have the ability of decreasing the contents of TG and LDL-C with moderate increase in the level of HDL-C [26–28]. Thus, our study suggested that the decreased levels of TC, TG, and LDL-C and the increased levels of HDL-C/LDL-C and HDL-C/TC after the simultaneous intakes of low cholesterol (0.04 g/kg total diet) and n-6/n-3 (5 : 1) may be in favor of reducing the incidence of associated metabolic diseases.

Besides, we found that the accumulation of various FAs was associated with the dietary intake of n-6/n-3 ratio, cholesterol, and even tissues. Although the blended oils fed to rats were with no EPA and DHA, these two PUFAs were detected in rats' plasma and four organs, especially high in brain and heart. This is because dietary ALA can convert into long-chain n-3 PUFA, such as EPA, DPA, and DHA due to the desaturation, elongation, and *β*-oxidation reactions [29, 30]. Increased levels of EPA, DHA, and ALA and decreased level of ARA were observed in rats' plasma after the intake of n-6/n-3 (5 : 1) and cholesterol (0.04 g/kg total diet) compared to rats fed with n-6/n-3 ratio of 9 : 1 and cholesterol of 0.057 g/kg total diet. The result suggested that negative correlations were found between the levels of DHA, ALA, or EPA and ARA in plasma. This may be due to the antagonistic effect between DHA, ALA, or EPA and ARA in absorption and metabolism. Moreover, as the n-6/n-3 PUFA ratio of the diet decreased, the proportion of n-3 PUFA increased in plasma, liver, and muscle, thus resulting in the decreased n-6/n-3 PUFA ratio. In accord with our results, Jeffery et al. [31] found that there were decreases in the levels of linoleic acid and ARA and an increase level of ALA in tissues as the n-6/n-3 ratio of the diet decreased. Umesha and Naidu [32] reported that dietary feeding of blended oils with n-6/n-3 ratio of 2.3–2.6 significantly increased ALA, EPA, and DHA content in serum, liver, and other tissues. We also found that the increasing cholesterol in diet increased the TMUFAs but decreased the TSFAs in rats' plasma and heart, which is in agreement with the results of Huang et al. [33] reporting an increase of 18:1n-9 after feeding rats with cholesterol. Therefore, due to the beneficial effect of n-3 PUFAs against n-6 PUFAs on metabolism, it may be helpful to prevent diseases by controlling dietary intakes of n-6/n-3 ratio (improving n-3 PUFAs) and cholesterol level at a low level.

## 5. Conclusions

High-fat diet increased the BWs of rats; however, the increase could be controlled by intakes of low cholesterol (0.04 g/kg total diet) and n-6/n-3 ratio (5 : 1). Besides, plasma lipid levels (TC and LDL-C) were also increased and HDL-C level was decreased under high-fat or high-cholesterol diet, but they were ameliorated in response to simultaneous intakes of low n-6/n-3 (5 : 1) and cholesterol (0.04 g/kg total diet). Likewise, the proportion of n-3 PUFA increased and the ratios of n-6/n-3 decreased in plasma, liver, and muscle as the n-6/n-3 PUFA ratio in the diet decreased.

## Figures and Tables

**Table 1:** The grouping of rats according to the diet of Chinese people.

	^b^The energy proportion of fat (%)	^c^The dose for oral administration of the blended oil in rats (mL/100 g·BW·d)	^d^The cholesterol level (g/kg total diet)	n-6/n-3
^a^EG 1	36.06	0.8	0.04	9 : 1
EG 2	36.06	0.8	0.057	9 : 1
EG 3	36.06	0.8	0.04	5 : 1
EG 4	36.06	0.8	0.057	5 : 1
CG 1	30	0.6	0.04	9 : 1
CG 2	30	0.6	0.057	9 : 1

^a^EG was experimental group and CG was control group. ^b^The energy of fat consumed in the urban areas (36.06%) and in the whole country (30%), respectively. ^c^Average intake of blended oils in rats converted from Chinese dietary consumption per day in the urban areas (40 g/60 kg·BW·d) and in the whole country (30 g/60 kg·BW·d), respectively. ^d^The cholesterol proportion of total diet in the whole country (0.04 g/kg total diet) and the urban areas (0.057 g/kg total diet), respectively.

**Table 2:** The components and nutrition compositions of basal chow and the amount of palm oil and cholesterol of each group.

Basal components (g/kg)	Groups					
	EG1	EG2	EG3	EG4	CG1	CG2
Corn flour	480	480	480	480	480	480
Soybean flour	210	210	210	210	210	210
Wheat bran	170	170	170	170	170	170
Wheat flour	50	50	50	50	50	50
Fish meal	50	50	50	50	50	50
CaH_2_PO_4_	15	15	15	15	15	15
Limestone power	15	15	15	15	15	15
Salt	5	5	5	5	5	5
Bean oil	6	6	6	6	6	6
Nutrient compositions (%)						
Carbohydrate	52.58					
Fat	3.50					
Protein	21.2					
Moisture	8.80					
Ash	6.78					
Fiber	2.80					
Additional components						
Palm oil	90	90	86	86	61	61
Cholesterol (mg/kg)	40	57	40	57	40	57

EG was experimental group and CG was control group. Different dietary treatments for groups: EG1 (0.8 mL blended oil, 9% palm oil, cholesterol of 0.04 g/kg total diet, and n-6/n-3 9 : 1); EG2 (0.8 mL blended oil, 9% palm oil, cholesterol of 0.057 g/kg total diet, and n-6/n-3 9 : 1); EG3 (0.8 mL blended oil, 8.6% palm oil, cholesterol of 0.04 g/kg total diet, and n-6/n-3 5 : 1); EG4 (0.8 mL blended oil, 8.6% palm oil, cholesterol of 0.057 g/kg total diet, and n-6/n-3 5 : 1); CG1 (0.6 mL blended oil, 6.1% palm oil, cholesterol of 0.04 g/kg total diet, and n-6/n-3 9 : 1); CG2 (0.6 mL blended oil, 6.1% palm oil, cholesterol of 0.057 g/kg total diet, and n-6/n-3 9 : 1). Different proportions of blended oil (g/100 g) for groups: EG1/EG2 (camellia oil 10 g, sunflower oil 74 g, and linseed oil 16 g); EG3/EG4 (camellia oil 18.6 g, sunflower oil 53.5 g, and linseed oil 27.9 g); CG1/CG2 (camellia oil 10.2 g, sunflower oil 73.5 g, and linseed oil 16.3 g).

**Table 3:** Main fatty acid compositions (% area) of four oils used.

Fatty acids	Palm oil	Camellia oil	Sunflower oil	Linseed oil
14:0	1.19	0.04	0.06	0.05
16:0	60.28	8.79	6.34	5.19
9c16:1	0.11	0.09	0.06	0.06
18:0	5.66	2.29	5.67	3.52
9t18:1	0.18	2.33	1.71	1.55
9c18:1 OA	23.98	76.46	19.12	20.35
11c18:1	1.41	ND	1.41	1.35
9c12c18:2n-6 LA	5.51	6.88	58.66	14.31
20:0	0.36	0.05	0.30	0.23
18:3n-6	ND	ND	ND	0.83
5c20:1	ND	0.02	ND	0.17
11c20:1	0.07	0.37	0.11	5.73
18:3n-3 ALA	0.21	0.28	0.21	34.65
21:0	ND	0.02	0.19	ND
22:0	0.05	0.01	0.56	0.07
22:1n-9	ND	0.03	ND	0.31
23:0	0.03	0.01	ND	ND
24:0	0.06	0.03	0.11	ND
TSFA	67.73	11.31	13.23	9.06
TMUFA	25.57	76.95	20.74	27.66
TPUFA	5.73	7.26	58.87	50.28
n-6/n-3	26.29	24.64	279.33	0.44

OA, oleic acid; LA, linoleic acid; ALA, alpha-linolenic acid; TSFAs, total saturated fatty acids, the sum of (C14:0 + C16:0 + C18:0 + C20:0 + C21:0 + C22:0 + C23:0 + C24:0 + ⋯); TMUFAs, total monounsaturated fatty acids, the sum of (C16:1 + C18:1 + C20:1 + C22:1 + ⋯); TPUFAs, total polyunsaturated fatty acids, the sum of (C18:2n-6 + C18:3n-6 + C18:3n-3 + ⋯). ND, not detected. Different dietary treatments for groups: EG1 (0.8 mL blended oil, 9% palm oil, cholesterol of 0.04 g/kg total diet, and n-6/n-3 9 : 1); EG2 (0.8 mL blended oil, 9% palm oil, cholesterol of 0.057 g/kg total diet, and n-6/n-3 9 : 1); EG3 (0.8 mL blended oil, 8.6% palm oil, cholesterol of 0.04 g/kg total diet, and n-6/n-3 5 : 1); EG4 (0.8 mL blended oil, 8.6% palm oil, cholesterol of 0.057 g/kg total diet, and n-6/n-3 5 : 1); CG1 (0.6 mL blended oil, 6.1% palm oil, cholesterol of 0.04 g/kg total diet, and n-6/n-3 9 : 1); CG2 (0.6 mL blended oil, 6.1% palm oil, cholesterol of 0.057 g/kg total diet, and n-6/n-3 9 : 1). Different proportions of blended oil (g/100 g) for groups: EG1/EG2 (camellia oil 10 g, sunflower oil 74 g, and linseed oil 16 g); EG3/EG4 (camellia oil 18.6 g, sunflower oil 53.5 g, and linseed oil 27.9 g); CG1/CG2 (camellia oil 10.2 g, sunflower oil 73.5 g, and linseed oil 16.3 g).

**Table 4:** The formulation design of blended oil intragastric gavage administered in each group.

Oil (g/100 g)	EG1/EG2	EG3/EG4	CG1/CG2
Camellia oil	10	18.6	10.2
Sunflower oil	74	53.5	73.5
Linseed oil	16	27.9	16.3
Total	100	100	100
Final fatty acid ratios administered to rats considering the oils in the chow			
S : M : P	1 : 1.22 : 1	1 : 1.19 : 1	1 : 1.21 : 1.03
n-6/n-3	9.12	5.04	9.07

EG was experimental group and CG was control group. S: total saturated fatty acids; M: total monounsaturated fatty acids; TPUFA: total polyunsaturated fatty acids. Different dietary treatments for groups: EG1 (0.8 mL blended oil, 9% palm oil, cholesterol of 0.04 g/kg total diet, and n-6/n-3 9 : 1); EG2 (0.8 mL blended oil, 9% palm oil, cholesterol of 0.057 g/kg total diet, and n-6/n-3 9 : 1); EG3 (0.8 mL blended oil, 8.6% palm oil, cholesterol of 0.04 g/kg total diet, and n-6/n-3 5 : 1); EG4 (0.8 mL blended oil, 8.6% palm oil, cholesterol of 0.057 g/kg total diet, and n-6/n-3 5 : 1); CG1 (0.6 mL blended oil, 6.1% palm oil, cholesterol of 0.04 g/kg total diet, and n-6/n-3 9 : 1); CG2 (0.6 mL blended oil, 6.1% palm oil, cholesterol of 0.057 g/kg total diet, and n-6/n-3 9 : 1). Different proportions of blended oil (g/100 g) for groups: EG1/EG2 (camellia oil 10 g, sunflower oil 74 g, and linseed oil 16 g); EG3/EG4 (camellia oil 18.6 g, sunflower oil 53.5 g, and linseed oil 27.9 g); CG1/CG2 (camellia oil 10.2 g, sunflower oil 73.5 g, and linseed oil 16.3 g).

**Table 5:** The body weights (g) of rats in weeks.

The week	EG1	EG2	EG3	EG4	CG1	CG2
0th	277 ± 26	277 ± 31	277 ± 40	277 ± 40	278 ± 32	277 ± 32
1st	307 ± 20	313 ± 25	313 ± 27	318 ± 32	316 ± 32	306 ± 19
2nd	349 ± 28	351 ± 23	349 ± 28	348 ± 32	343 ± 35	330 ± 19
3rd	385 ± 34	386 ± 21	361 ± 32	373 ± 38	370 ± 38	362 ± 22
4th	412 ± 30^c^	410 ± 22^c^	368 ± 31^a^	401 ± 30^bc^	390 ± 29^ac^	380 ± 27^ab^
5th	426 ± 32^c^	429 ± 22^c^	374 ± 29^a^	423 ± 33^c^	413 ± 30^bc^	391 ± 25^ab^
6th	447 ± 27^c^	435 ± 21^c^	378 ± 25^a^	444 ± 26^c^	424 ± 29^bc^	393 ± 30^ab^
7th	458 ± 32^c^	450 ± 25^c^	378 ± 26^a^	456 ± 31^c^	439 ± 31^bc^	412 ± 29^ab^
8th	466 ± 30^c^	463 ± 26^c^	375 ± 28^a^	460 ± 31^c^	452 ± 29^bc^	423 ± 27^b^
9th	481 ± 25^c^	474 ± 28^c^	376 ± 26^a^	465 ± 28^c^	462 ± 30^bc^	431 ± 29^bc^

Values presented as means (*n* = 12) ± SD. Mean values within each row followed by different letters (a, b, c) are significantly (*P* < 0.05) different. Different dietary treatments for groups: EG1 (0.8 mL blended oil, 9% palm oil, cholesterol of 0.04 g/kg total diet, and n-6/n-3 9 : 1); EG2 (0.8 mL blended oil, 9% palm oil, cholesterol of 0.057 g/kg total diet, and n-6/n-3 9 : 1); EG3 (0.8 mL blended oil, 8.6% palm oil, cholesterol of 0.04 g/kg total diet, and n-6/n-3 5 : 1); EG4 (0.8 mL blended oil, 8.6% palm oil, cholesterol of 0.057 g/kg total diet, and n-6/n-3 5 : 1); CG1 (0.6 mL blended oil, 6.1% palm oil, cholesterol of 0.04 g/kg total diet, and n-6/n-3 9 : 1); CG2 (0.6 mL blended oil, 6.1% palm oil, cholesterol of 0.057 g/kg total diet, and n-6/n-3 9 : 1). Different proportions of blended oil (g/100 g) for groups: EG1/EG2 (camellia oil 10 g, sunflower oil 74 g, and linseed oil 16 g); EG3/EG4 (camellia oil 18.6 g, sunflower oil 53.5 g, and linseed oil 27.9 g); CG1/CG2 (camellia oil 10.2 g, sunflower oil 73.5 g, and linseed oil 16.3 g).

**Table 6:** The biochemical indices of TC, TG, HDL-C, and LDL-C (mmol/L) and the ratios of HDL-C/LDL-C and HDL-C/TC.

		EG 1	EG 2	EG 3	EG 4	CG 1	CG 2
TC	B	1.16 ± 0.11	1.10 ± 0.13^(a)^	1.16 ± 0.20	1.16 ± 0.15^(a)^	1.12 ± 0.09	1.12 ± 0.12^(a)^
	A	1.28 ± 0.30^a^	1.73 ± 0.33^b(b)^	1.28 ± 0.21^a^	2.06 ± 0.24^c(b)^	1.23 ± 0.21^a^	1.58 ± 0.30^b(b)^
TG	B	0.48 ± 0.09	0.41 ± 0.01^(b)^	0.44 ± 0.03	0.53 ± 0.03^(b)^	0.51 ± 0.04	0.48 ± 0.08
	A	0.43 ± 0.06^bc^	0.35 ± 0.05^a(a)^	0.46 ± 0.08^c^	0.38 ± 0.04^ab(a)^	0.45 ± 0.12^bc^	0.44 ± 0.08^bc^
HDL-C	B	0.70 ± 0.07	0.69 ± 0.07	0.73 ± 0.07^(b)^	0.78 ± 1.04^(a)^	0.70 ± 0.06^(b)^	0.67 ± 0.10^(b)^
	A	0.61 ± 0.09^b^	0.61 ± 0.12^b^	0.63 ± 0.10^b(a)^	0.99 ± 0.11^c(b)^	0.54 ± 0.08^ab(a)^	0.50 ± 0.04^a(a)^
LDL-C	B	0.30 ± 0.04^(a)^	0.25 ± 0.07^(a)^	0.28 ± 0.07^(a)^	0.30 ± 0.09^(a)^	0.30 ± 0.07	0.28 ± 0.09
	A	0.39 ± 0.07^a(b)^	0.42 ± 0.07^a(b)^	0.38 ± 0.06^a(b)^	0.67 ± 0.11^b(b)^	0.36 ± 0.05^a^	0.39 ± 0.08^a^
HDL-C/LDL-C	B	2.34 ± 0.23^(b)^	3.12 ± 0.24^(b)^	2.00 ± 0.53^(b)^	2.73 ± 0.65^(b)^	2.04 ± 0.48	2.69 ± 0.32^(b)^
	A	1.52 ± 0.26^a(a)^	1.47 ± 0.29^a(a)^	2.24 ± 0.26^b(a)^	1.53 ± 0.26^a(a)^	2.11 ± 0.49^b^	1.97 ± 0.46^b(a)^
HDL-C/TC	B	0.61 ± 0.03^(b)^	0.65 ± 0.05^(b)^	0.65 ± 0.08^(b)^	0.68 ± 0.08^(b)^	0.67 ± 0.04^(b)^	0.67 ± 0.02^(b)^
	A	0.48 ± 0.08^b(a)^	0.36 ± 0.04^a(a)^	0.46 ± 0.07^b(a)^	0.45 ± 0.08^b(a)^	0.40 ± 0.11^ab(a)^	0.37 ± 0.09^a(a)^

Values presented as means (*n* = 12) ± SD. B and A represented before and after treatment, respectively. TC, total cholesterol; TG, triacylglycerols; VLDL-C, very low density lipoprotein cholesterol; HDL-C, high density lipoprotein cholesterol; LDL-C, low density lipoprotein cholesterol. Mean values within each row followed by different letters (a, b, c) are significantly (*P* < 0.05) different. ^(a)^versus B and ^(b)^versus A. Different dietary treatments for groups: EG1 (0.8 mL blended oil, 9% palm oil, cholesterol of 0.04 g/kg total diet, and n-6/n-3 9 : 1); EG2 (0.8 mL blended oil, 9% palm oil, cholesterol of 0.057 g/kg total diet, and n-6/n-3 9 : 1); EG3 (0.8 mL blended oil, 8.6% palm oil, cholesterol of 0.04 g/kg total diet, and n-6/n-3 5 : 1); EG4 (0.8 mL blended oil, 8.6% palm oil, cholesterol of 0.057 g/kg total diet, and n-6/n-3 5 : 1); CG1 (0.6 mL blended oil, 6.1% palm oil, cholesterol of 0.04 g/kg total diet, and n-6/n-3 9 : 1); CG2 (0.6 mL blended oil, 6.1% palm oil, cholesterol of 0.057 g/kg total diet, and n-6/n-3 9 : 1). Different proportions of blended oil (g/100 g) for groups: EG1/EG2 (camellia oil 10 g, sunflower oil 74 g, and linseed oil 16 g); EG3/EG4 (camellia oil 18.6 g, sunflower oil 53.5 g, and linseed oil 27.9 g); CG1/CG2 (camellia oil 10.2 g, sunflower oil 73.5 g, and linseed oil 16.3 g).

**Table 7:** The correlation analyses of biochemical indices (HDL-C/LDL-C, TC, TG, and LDL-C).

Dependent variable	Independent variable	Correlation coefficient	*P* value
HDL-C/LDL-C	TC	−0.423^*∗∗*^	0.000
	TG	−0.539^*∗*^	0.010
	LDL-C	−0.634^*∗∗*^	0.000
TC	TG	0.210^*∗*^	0.042
	LDL-C	−0.253^*∗*^	0.013

^*∗∗*^ *P* lower than 0.01 (^*∗∗*^*P* < 0.01) was very significantly different, and ^*∗*^*P* lower than 0.05 (^*∗∗*^*P* < 0.05) was significantly different; TC, total cholesterol; TG, triacylglycerols; VLDL-C, very low density lipoprotein cholesterol; HDL-C, high density lipoprotein cholesterol; LDL-C, low density lipoprotein cholesterol.

**Table 8:** Main fatty acid compositions (% area) in rats' plasma after 60 days' treatment.

Fatty acids	EG1	EG2	EG3	EG4	CG1	CG2
14:0	0.21 ± 0.06^ab^	0.20 ± 0.07^ab^	0.16 ± 0.06^a^	0.21 ± 0.04^b^	0.21 ± 0.04^ab^	0.23 ± 0.06^b^
16:0	21.32 ± 2.08^ab^	22.06 ± 1.07^ac^	21.61 ± 1.10^ab^	20.72 ± 2.7^a^	23.21 ± 1.0^c^	22.68 ± 1.0^bc^
9c16:1	0.21 ± 0.05^a^	0.16 ± 0.08^a^	0.15 ± 0.03^a^	0.18 ± 0.05^a^	0.16 ± 0.04^a^	0.46 ± 0.40^b^
18:0	18.62 ± 2.3^c^	17.40 ± 1.31^c^	19.20 ± 3.14^c^	13.38 ± 4.24^a^	16.29 ± 2.50^bc^	14.63 ± 1.94^ab^
9t18:1	0.10 ± 0.02^b^	0.11 ± 0.06^b^	0.10 ± 0.01^b^	0.10 ± 0.02^b^	0.08 ± 0.01^a^	0.09 ± 0.01^ab^
9c18:1 OA	9.30 ± 3.06^a^	10.77 ± 1.61^a^	11.14 ± 3.05^a^	13.38 ± 1.94^c^	11.46 ± 2.29^ab^	13.53 ± 2.41^bc^
11c18:1	1.21 ± 0.22^a^	1.53 ± 0.31^b^	1.12 ± 0.24^a^	1.65 ± 0.27^b^	1.76 ± 0.42^bc^	1.97 ± 0.32^c^
9c12c18:2 LA	19.47 ± 3.76^a^	20.59 ± 7.03^a^	25.10 ± 1.56^b^	25.23 ± 3.22^b^	24.53 ± 1.92^b^	25.13 ± 2.15^b^
20:0	0.21 ± 0.06^c^	0.19 ± 0.02^bc^	0.17 ± 0.02^b^	0.16 ± 0.02^b^	0.13 ± 0.02^a^	0.13 ± 0.04^a^
18:3n-6	0.13 ± 0.06^ab^	0.11 ± 0.01^a^	0.17 ± 0.07^bc^	0.17 ± 0.09^bc^	0.15 ± 0.04^abc^	0.19 ± 0.06^c^
5c20:1	0.25 ± 0.04^b^	0.25 ± 0.04^b^	0.17 ± 0.05^a^	0.20 ± 0.05^a^	0.20 ± 0.07^a^	0.18 ± 0.02^a^
11c20:1	0.27 ± 0.12^a^	0.36 ± 0.10^a^	0.65 ± 0.25^a^	1.66 ± 2.09^b^	0.44 ± 0.18^a^	0.61 ± 0.20^a^
18:3n-3 ALA	0.39 ± 0.08^ab^	0.39 ± 0.08^ab^	0.43 ± 0.05^ab^	0.47 ± 0.10^b^	0.35 ± 0.06^a^	0.38 ± 0.04^ab^
20:2n-6	0.35 ± 0.07^c^	0.35 ± 0.05^bc^	0.23 ± 0.06^a^	0.31 ± 0.07^bc^	0.36 ± 0.10^c^	0.29 ± 0.03^b^
22:0	0.11 ± 0.06^b^	0.06 ± 0.01^a^	0.07 ± 0.03^ab^	0.16 ± 0.05^c^	0.18 ± 0.04^c^	0.16 ± 0.03^c^
20:4n-6 ARA	9.67 ± 1.90^bc^	11.08 ± 1.89^c^	7.45 ± 1.72^a^	7.87 ± 1.83^a^	10.03 ± 1.82^c^	9.65 ± 1.75^bc^
22:2n-6	0.37 ± 0.08^ab^	0.37 ± 0.23^ab^	0.26 ± 0.07^a^	0.37 ± 0.13^b^	0.33 ± 0.10^ab^	0.31 ± 0.09^ab^
20:5n-3 EPA	0.28 ± 0.02^a^	0.39 ± 0.06^b^	0.52 ± 0.05^cd^	0.62 ± 0.09^d^	0.42 ± 0.05^bc^	0.49 ± 0.06^bc^
24:0	0.11 ± 0.05^b^	0.06 ± 0.03^a^	0.05 ± 0.02^a^	0.06 ± 0.02^a^	0.05 ± 0.01^a^	0.04 ± 0.01^a^
22:3n-3	0.52 ± 0.23^b^	0.56 ± 0.13^b^	0.57 ± 0.36^b^	0.56 ± 0.28^b^	0.38 ± 0.26^ab^	0.18 ± 0.04^a^
22:4n-6	0.27 ± 0.04^d^	0.25 ± 0.05^cd^	0.15 ± 0.05^a^	0.19 ± 0.05^ab^	0.19 ± 0.05^ab^	0.22 ± 0.05^bc^
22:5n-6	0.68 ± 0.15^bc^	0.69 ± 0.20^bc^	0.74 ± 0.24^bc^	0.82 ± 0.19^c^	0.66 ± 0.15^b^	0.34 ± 0.11^a^
22:5n-3 DPA	0.50 ± 0.23	0.51 ± 0.16	0.67 ± 0.27	0.54 ± 0.12	0.45 ± 0.26	0.58 ± 0.18
22:6n-3 DHA	3.29 ± 0.42^ac^	3.36 ± 0.33^ac^	3.72 ± 0.69^c^	3.51 ± 0.71^bc^	3.02 ± 0.18^a^	3.07 ± 0.22^ab^
TSFAs	41.33 ± 3.21^bc^	40.82 ± 1.30^bc^	41.87 ± 2.67^c^	35.55 ± 2.23^a^	40.95 ± 2.47^bc^	38.55 ± 2.90^ab^
TMUFAs	11.48 ± 3.45^a^	13.41 ± 1.78^ab^	13.50 ± 3.34^ab^	17.78 ± 2.89^d^	14.25 ± 2.81^bc^	17.01 ± 2.84^cd^
TPUFAs	41.50 ± 1.09	40.20 ± 2.35	42.30 ± 1.40	42.49 ± 1.45	42.61 ± 1.15	42.20 ± 1.08
n-6 PUFAs	36.51 ± 1.14^a^	36.97 ± 1.21^ab^	36.49 ± 1.26^a^	36.79 ± 1.01^a^	37.95 ± 1.18^c^	37.37 ± 1.29^bc^
n-3 PUFAs	4.99 ± 0.69^a^	5.23 ± 0.54^ab^	5.80 ± 0.99^b^	5.70 ± 0.99^b^	4.66 ± 0.72^a^	4.83 ± 0.64^a^
n-6/n-3 ratio	7.32 ± 0.12^b^	7.07 ± 0.18^ab^	6.29 ± 0.13^a^	6.45 ± 0.10^a^	8.14 ± 0.19^c^	7.72 ± 0.15^bc^
S/M/P	1.09 : 0.30 : 1	1.01 : 0.33 : 1	1 : 0.32 : 1.01	1 : 0.50 : 1.19	1 : 0.35 : 1.04	1 : 0.44 : 1.09

Values presented as means (*n* = 12) ± SD. Mean values within each row followed by different letters (a, b, c, etc.) are significantly (*P* < 0.05) different. OA, oleic acid; LA, linoleic acid; ALA, alpha-linolenic acid; EPA, eicosapntemacnioc acid; ARA, arachidonic acid; DPA, docosapentaenoic acid; DHA, docosahexenoic acid; TSFAs, total saturated fatty acids, the sum of (C14:0 + C16:0 + C18:0 + C20:0 + C22:0 +C24:0 + ⋯); TMUFAs, total monounsaturated fatty acids, the sum of (C16:1 + C18:1 + C20:1 + ⋯); TPUFAs, total polyunsaturated fatty acids, the sum of (C18:2n-6 + C18:3n-6 + C18:3n-3 + C20:2n-6 + 20:4n-6 + C22:2n-6 + C20:5n-3 + C22:3n-3 + C22:4n-6 + C22:5n-6 + C22:5n-3 + C22:6n-3 + ⋯); S/M/P: TSFA/TMUFA/TPUFA. Different dietary treatments for groups: EG1 (0.8 mL blended oil, 9% palm oil, cholesterol of 0.04 g/kg total diet, and n-6/n-3 9 : 1); EG2 (0.8 mL blended oil, 9% palm oil, cholesterol of 0.057 g/kg total diet, and n-6/n-3 9 : 1); EG3 (0.8 mL blended oil, 8.6% palm oil, cholesterol of 0.04 g/kg total diet, and n-6/n-3 5 : 1); EG4 (0.8 mL blended oil, 8.6% palm oil, cholesterol of 0.057 g/kg total diet, and n-6/n-3 5 : 1); CG1 (0.6 mL blended oil, 6.1% palm oil, cholesterol of 0.04 g/kg total diet, and n-6/n-3 9 : 1); CG2 (0.6 mL blended oil, 6.1% palm oil, cholesterol of 0.057 g/kg total diet, and n-6/n-3 9 : 1). Different proportions of blended oil (g/100 g) for groups: EG1/EG2 (camellia oil 10 g, sunflower oil 74 g, and linseed oil 16 g); EG3/EG4 (camellia oil 18.6 g, sunflower oil 53.5 g, and linseed oil 27.9 g); CG1/CG2 (camellia oil 10.2 g, sunflower oil 73.5 g, and linseed oil 16.3 g).

**Table 9:** Fatty acid compositions (% area) in rats' liver and muscle after 60 days' treatment.

Fatty acids	EG1	EG2	EG3	EG4	CG1	CG2
Fatty acid compositions (% area) in rats' liver						
16:0	17.41 ± 1.08^a^	18.19 ± 1.54^ab^	17.72 ± 0.82^a^	17.69 ± 2.90^a^	19.59 ± 1.86^b^	18.67 ± 0.90^ab^
9c16:1	0.43 ± 0.19^a^	0.58 ± 0.36^bc^	0.40 ± 0.12^a^	0.61 ± 0.37^bc^	0.78 ± 0.52^c^	0.50 ± 0.31^ab^
18:0	6.41 ± 1.42^ab^	6.49 ± 1.42^ab^	6.51 ± 2.08^ab^	7.02 ± 5.66^ab^	4.92 ± 2.42^a^	7.77 ± 3.51^b^
9t18:1	0.23 ± 0.02	0.23 ± 0.02	0.24 ± 0.02	0.23 ± 0.04	0.23 ± 0.02	0.22 ± 0.02
11t18:1	0.19 ± 0.02^a^	0.19 ± 0.02^a^	0.22 ± 0.02^b^	0.20 ± 0.04^ab^	0.18 ± 0.01^a^	0.19 ± 0.02^a^
9c18:1 OA	25.09 ± 2.40^ab^	25.02 ± 1.43^ab^	24.79 ± 2.40^ab^	25.06 ± 6.57^ab^	27.15 ± 3.79^b^	23.15 ± 4.52^a^
11c18:1	1.29 ± 0.18^ab^	1.37 ± 0.30^b^	1.07 ± 0.13^a^	1.37 ± 0.32^b^	1.69 ± 0.38^c^	1.45 ± 0.33^b^
9c12c18:2 LA	30.58 ± 1.33^ab^	29.47 ± 1.80^ab^	31.00 ± 1.32^b^	29.07 ± 2.47^a^	30.08 ± 2.28^a^	29.59 ± 2.54^ab^
20:0	0.11 ± 0.02^b^	0.10 ± 0.03^ab^	0.10 ± 0.03^ab^	0.11 ± 0.03^b^	0.09 ± 0.02^a^	0.11 ± 0.33^b^
18:3n-6	0.41 ± 0.10^a^	0.39 ± 0.06^a^	0.77 ± 0.23^b^	0.40 ± 0.07^a^	0.37 ± 0.07^a^	0.36 ± 0.08^a^
11c20:1	0.48 ± 0.10^c^	0.43 ± 0.11^bc^	0.28 ± 0.09^a^	0.35 ± 0.11^ab^	0.43 ± 0.13^bc^	0.36 ± 0.11^ab^
18:3n-3 ALA	1.40 ± 0.18^a^	1.43 ± 0.28^a^	3.19 ± 0.82^b^	2.80 ± 0.92^b^	1.45 ± 0.23^a^	1.12 ± 0.33^a^
20:2n-6	0.54 ± 0.12^d^	0.48 ± 0.10^cd^	0.27 ± 0.10^a^	0.38 ± 0.08^b^	0.49 ± 0.14^cd^	0.40 ± 0.07^bc^
22:0	0.04 ± 0.01	0.04 ± 0.01	0.04 ± 0.02	0.05 ± 0.02	0.04 ± 0.01	0.05 ± 0.01
20:3n-6	1.27 ± 0.22^c^	1.20 ± 0.20^bc^	1.57 ± 0.24^d^	1.00 ± 0.18^b^	0.99 ± 0.16^a^	1.04 ± 0.25^ab^
20:3n-3	0.04 ± 0.01	0.04 ± 0.00^ab^	0.05 ± 0.01^b^	0.07 ± 0.03^c^	0.05 ± 0.01^b^	0.03 ± 0.01^a^
20:4n-6 ARA	5.31 ± 0.55^bc^	5.70 ± 0.70^c^	3.93 ± 0.80^a^	4.31 ± 0.53^ab^	5.68 ± 0.71^c^	6.15 ± 0.73^c^
20:5n-3 EPA	0.54 ± 0.06^a^	0.53 ± 0.05^a^	0.74 ± 0.07^c^	0.70 ± 0.05^c^	0.53 ± 0.08^a^	0.61 ± 0.05^ab^
22:3n-3	0.22 ± 0.06^ab^	0.28 ± 0.16^ac^	0.21 ± 0.15^a^	0.32 ± 0.09^ac^	0.34 ± 0.18^bc^	0.36 ± 0.15^c^
22:4n-6	0.46 ± 0.12^d^	0.36 ± 0.08^c^	0.21 ± 0.08^a^	0.24 ± 0.06^ab^	0.31 ± 0.08^bc^	0.33 ± 0.10^c^
22:5n-6	0.18 ± 0.05^b^	0.16 ± 0.04^ab^	0.14 ± 0.05^ab^	0.13 ± 0.07^a^	0.13 ± 0.03^a^	0.12 ± 0.03^a^
22:5n-3 DPA	1.23 ± 0.27	1.09 ± 0.22	1.07 ± 0.29	1.16 ± 0.32	1.04 ± 0.18	1.17 ± 0.37
22:6n-3 DHA	3.93 ± 0.43	3.96 ± 0.61	3.38 ± 0.57	3.68 ± 0.73	3.08 ± 0.72	3.85 ± 0.99
TSFAs	24.70 ± 1.40^a^	25.75 ± 1.22^ab^	25.12 ± 2.33^a^	25.56 ± 3.16^ab^	25.47 ± 2.71^ab^	27.32 ± 3.24^b^
TMUFAs	27.74 ± 2.62^ab^	27.84 ± 1.81^ab^	27.00 ± 2.43^ab^	27.86 ± 6.78^ab^	30.45 ± 4.42^b^	25.89 ± 5.02^a^
TPUFAs	46.25 ± 2.18^b^	45.07 ± 2.21^ab^	46.18 ± 1.17^b^	45.10 ± 4.52^ab^	42.74 ± 3.21^a^	45.54 ± 2.78^b^
n-6 PUFAs	38.91 ± 1.68^b^	37.75 ± 1.78^ab^	37.94 ± 0.96^ab^	36.46 ± 3.07^a^	36.27 ± 2.52^a^	38.41 ± 1.99^b^
n-3 PUFAs	7.35 ± 0.61^bc^	7.32 ± 0.60^bc^	8.63 ± 0.57^d^	8.63 ± 1.59^d^	6.48 ± 0.82^a^	7.13 ± 1.04^ab^
n-6/n-3 ratio	5.32 ± 0.34^bc^	5.17 ± 0.30^b^	4.40 ± 0.33^a^	4.28 ± 0.43^a^	5.64 ± 0.46^c^	5.50 ± 0.86^bc^
S/M/P	1 : 1.12 : 1.67	1 : 1.08 : 1.75	1 : 1.07 : 1.84	1 : 1.09 : 1.76	1 : 1.20 : 1.68	1 : 0.95 : 1.67

Fatty acid compositions (% area) in rats' muscle						
16:0	23.30 ± 1.90^bd^	22.45 ± 1.62^ab^	22.06 ± 0.90^a^	22.64 ± 1.2^ac^	24.00 ± 1.05^d^	23.74 ± 1.22^cd^
18:0	4.05 ± 2.42	3.96 ± 1.28	4.04 ± 0.69	3.52 ± 0.86	4.00 ± 1.36	4.07 ± 0.64
9c18:1 OA	32.57 ± 7.02	33.96 ± 4.12	35.12 ± 2.12	35.90 ± 2.33	34.28 ± 3.84	34.04 ± 2.35
11c18:1	2.10 ± 0.34^c^	2.17 ± 0.19^c^	1.57 ± 0.15^a^	1.75 ± 0.11^ab^	1.68 ± 0.78^a^	2.02 ± 0.11^bc^
9c12c18:2 LA	24.81 ± 2.04^cd^	25.28 ± 2.45^d^	22.64 ± 1.77^ab^	21.42 ± 1.51^a^	22.08 ± 2.04^a^	23.16 ± 1.92^bc^
18:3n-6	0.03 ± 0.01	0.03 ± 0.01	0.04 ± 0.01	0.03 ± 0.01	0.03 ± 0.01	0.03 ± 0.01
18:3n-3 ALA	1.42 ± 0.29^a^	1.40 ± 0.22^a^	2.93 ± 0.56^c^	2.53 ± 0.4^b^	1.27 ± 0.24^a^	1.28 ± 0.20^a^
20:2n-6	0.15 ± 0.26	0.08 ± 0.01	0.07 ± 0.01	0.07 ± 0.01	0.07 ± 0.02	0.07 ± 0.01
20:3n-6	0.25 ± 0.26^b^	0.20 ± 0.15^ab^	0.22 ± 0.11^ab^	0.12 ± 0.08^ab^	0.17 ± 0.14^ab^	0.09 ± 0.07^a^
20:4n-6 ARA	1.34 ± 0.47	1.60 ± 0.46	1.16 ± 0.40	0.90 ± 0.10	1.63 ± 0.16	1.50 ± 0.41
20:5n-3 EPA	0.05 ± 0.04	0.06 ± 0.02	0.06 ± 0.02	0.05 ± 0.03	0.06 ± 0.04	0.06 ± 0.02
22:4n-6	0.07 ± 0.07	0.06 ± 0.05	0.04 ± 0.02	0.04 ± 0.04	0.05 ± 0.05	0.04 ± 0.02
22:5n-3 DPA	0.12 ± 0.07	0.19 ± 0.06	0.21 ± 0.07	0.17 ± 0.03	0.16 ± 0.07	0.18 ± 0.04
22:6n-3 DHA	0.76 ± 0.26	0.80 ± 0.20	0.88 ± 0.23	0.90 ± 0.24	0.81 ± 0.29	0.75 ± 0.27
TSFAs	28.73 ± 4.01^ab^	27.87 ± 2.45^ab^	27.61 ± 1.40^a^	27.67 ± 1.50^a^	29.62 ± 1.87^b^	29.43 ± 1.45^ab^
TMUFAs	38.76 ± 3.18^a^	40.02 ± 4.41^ab^	42.85 ± 2.03^bc^	44.41 ± 3.11^c^	42.17 ± 4.81^ac^	41.71 ± 3.08^ac^
TPUFAs	28.92 ± 2.89	29.87 ± 2.60	28.33 ± 1.78	26.14 ± 2.99	26.38 ± 3.73	27.17 ± 2.95
n-6 PUFAs	26.56 ± 2.84^bc^	27.35 ± 2.79^c^	24.21 ± 1.52^ab^	22.60 ± 2.40^a^	24.03 ± 2.06^ab^	24.91 ± 1.39^ab^
n-3 PUFAs	2.32 ± 0.23^a^	2.45 ± 0.22^a^	4.10 ± 0.22^b^	3.51 ± 0.23^b^	2.29 ± 0.16^a^	2.22 ± 0.17^a^
n-6/n-3 ratio	11.43 ± 1.62^b^	11.15 ± 1.58^b^	5.91 ± 1.26^a^	6.44 ± 1.66^a^	10.47 ± 1.70^b^	11.23 ± 1.48^b^
S/M/P	1 : 1.35 : 1.0	1 : 1.44 : 1.07	1 : 1.55 : 1.02	1 : 1.60 : 0.94	1 : 1.42 : 0.89	1 : 1.42 : 0.92

Values presented as means (*n* = 12) ± SD. Mean values within each row followed by different letters (a, b, c, etc.) are significantly (*P* < 0.05) different. OA, oleic acid; LA, linoleic acid; ALA, alpha-linolenic acid; EPA, eicosapntemacnioc acid; ARA, arachidonic acid; DPA, docosapentaenoic acid; DHA, docosahexenoic acid; TSFAs, total saturated fatty acids, the sum of (C16:0 + C18:0 + C20:0 + C22:0 + ⋯); TMUFAs, total monounsaturated fatty acids, the sum of (C16:1 + C18:1 + C20:1 + C22:1 + C24:1 + ⋯); TPUFAs, total polyunsaturated fatty acids, the sum of (C18:2n-6 + C18:3n-3 + C20:2n-6 + C20:3n-6 + C20:4n-6 + C22:2n-6 + C20:5n-3 + C22:4n-6 + C22:5n-6 + C22:5n-3 + 22:6n-3 + ⋯); S/M/P: TSFAs/TMUFAs/TPUFAs. Different dietary treatments for groups: EG1 (0.8 mL blended oil, 9% palm oil, cholesterol of 0.04 g/kg total diet, and n-6/n-3 9 : 1); EG2 (0.8 mL blended oil, 9% palm oil, cholesterol of 0.057 g/kg total diet, and n-6/n-3 9 : 1); EG3 (0.8 mL blended oil, 8.6% palm oil, cholesterol of 0.04 g/kg total diet, and n-6/n-3 5 : 1); EG4 (0.8 mL blended oil, 8.6% palm oil, cholesterol of 0.057 g/kg total diet, and n-6/n-3 5 : 1); CG1 (0.6 mL blended oil, 6.1% palm oil, cholesterol of 0.04 g/kg total diet, and n-6/n-3 9 : 1); CG2 (0.6 mL blended oil, 6.1% palm oil, cholesterol of 0.057 g/kg total diet, and n-6/n-3 9 : 1). Different proportions of blended oil (g/100 g) for groups: EG1/EG2 (camellia oil 10 g, sunflower oil 74 g, and linseed oil 16 g); EG3/EG4 (camellia oil 18.6 g, sunflower oil 53.5 g, and linseed oil 27.9 g); CG1/CG2 (camellia oil 10.2 g, sunflower oil 73.5 g, and linseed oil 16.3 g).

**Table 10:** Fatty acid compositions (% area) in rats' brain and heart after 60 days' treatment.

Fatty acids	EG1	EG2	EG3	EG4	CG1	CG2
Fatty acid compositions (% area) in rats' brain						
16:00	21.53 ± 1.35	21.68 ± 1.16	21.36 ± 0.76	21.48 ± 1.05	21.09 ± 1.06	20.83 ± 0.79
9c16:1	0.38 ± 0.03^ab^	0.34 ± 0.03^a^	0.37 ± 0.04^ab^	0.36 ± 0.04^ab^	0.38 ± 0.03^b^	0.38 ± 0.03^b^
18:00	20.69 ± 0.60^a^	21.31 ± 0.46^b^	20.45 ± 0.52^a^	20.51 ± 0.56^a^	20.54 ± 0.42^a^	20.46 ± 0.44^a^
9t18:1	0.18 ± 0.04^b^	0.13 ± 0.12^a^	0.14 ± 0.03^a^	0.14 ± 0.02^a^	0.16 ± 0.03^ab^	0.19 ± 0.04^b^
9c18:1 OA	20.01 ± 1.26^ab^	19.22 ± 1.26^a^	19.95 ± 1.01^ab^	20.64 ± 1.11^b^	20.85 ± 1.05^bc^	21.68 ± 1.12^c^
11c18:1	3.86 ± 0.36^abc^	3.78 ± 0.31^ab^	3.73 ± 0.28^a^	4.04 ± 0.38^bc^	4.15 ± 0.31^c^	4.14 ± 0.33^c^
9c12c18:2 LA	1.20 ± 0.24^a^	1.08 ± 0.11^a^	1.45 ± 0.27^b^	1.15 ± 0.23^a^	1.13 ± 0.08^a^	1.13 ± 0.16^a^
20:00	0.40 ± 0.10^bc^	0.29 ± 0.09^a^	0.34 ± 0.08^ab^	0.34 ± 0.08^ab^	0.39 ± 0.09^bc^	0.44 ± 0.10^c^
5c20:1	0.06 ± 0.02^ab^	0.05 ± 0.01^a^	0.04 ± 0.01^a^	0.05 ± 0.00^ab^	0.05 ± 0.01^ab^	0.06 ± 0.01^b^
11c20:1	2.18 ± 0.69^ab^	1.73 ± 0.62^a^	2.13 ± 0.58^ab^	2.18 ± 0.64^ab^	2.34 ± 0.59^b^	2.50 ± 0.64^b^
18:3n-3 ALA	0.60 ± 0.18	0.54 ± 0.16	0.57 ± 0.15	0.59 ± 0.11	0.65 ± 0.16	0.69 ± 0.15
20:2n-6	0.23 ± 0.05^bc^	0.19 ± 0.04^a^	0.26 ± 0.04^c^	0.23 ± 0.06^bc^	0.22 ± 0.04^b^	0.21 ± 0.03^ab^
22:00	0.28 ± 0.05^bc^	0.23 ± 0.06^a^	0.24 ± 0.04^ab^	0.24 ± 0.03^ab^	0.29 ± 0.06^cd^	0.33 ± 0.05^d^
20:3n-6	0.47 ± 0.02^ab^	0.45 ± 0.03^a^	0.63 ± 0.03^c^	0.47 ± 0.03^ab^	0.48 ± 0.04^b^	0.46 ± 0.03^ab^
22:1n-9	0.24 ± 0.07^bc^	0.18 ± 0.06^a^	0.22 ± 0.06^ab^	0.21 ± 0.04^ab^	0.25 ± 0.06^bc^	0.27 ± 0.06^c^
20:4n-6 ARA	9.87 ± 0.55^ab^	10.28 ± 0.48^b^	9.81 ± 0.46^a^	9.66 ± 0.66^a^	9.53 ± 0.54^a^	9.45 ± 0.51^a^
22:2n-6	0.07 ± 0.05^ab^	0.04 ± 0.02^a^	0.06 ± 0.04^ab^	0.06 ± 0.02^ab^	0.08 ± 0.03^b^	0.08 ± 0.03^b^
20:5n-3 EPA	0.23 ± 0.04^ab^	0.19 ± 0.03^a^	0.23 ± 0.04^ab^	0.22 ± 0.02^ab^	0.24 ± 0.05^bc^	0.27 ± 0.06^c^
24:1n-9	0.12 ± 0.04^ab^	0.10 ± 0.03^a^	0.11 ± 0.02	0.11 ± 0.01^ab^	0.13 ± 0.03^bc^	0.15 ± 0.05^c^
22:4n-6	2.86 ± 0.19	2.81 ± 0.20	2.73 ± 0.19	2.76 ± 0.22	2.75 ± 0.19	2.77 ± 0.16
22:5n-6	0.48 ± 0.08^c^	0.47 ± 0.12^c^	0.31 ± 0.04^a^	0.38 ± 0.07^b^	0.39 ± 0.06^b^	0.43 ± 0.07^bc^
22:5n-3 DPA	0.17 ± 0.02^ab^	0.15 ± 0.01^a^	0.22 ± 0.02^c^	0.18 ± 0.02^b^	0.16 ± 0.02^a^	0.17 ± 0.03^ab^
22:6n-3 DHA	12.69 ± 1.00^bc^	13.66 ± 0.96^d^	13.26 ± 0.99^cd^	12.66 ± 0.73^bc^	12.34 ± 0.86^b^	11.49 ± 0.95^a^
TSFAs	43.33 ± 1.34^ab^	43.91 ± 1.33^b^	42.87 ± 0.93^a^	43.00 ± 1.10^ab^	42.76 ± 1.18^a^	42.53 ± 1.06^a^
TMUFAs	27.08 ± 2.33^ab^	25.61 ± 2.21^a^	26.79 ± 1.88^ab^	27.82 ± 2.12^bc^	28.41 ± 1.99^bc^	29.47 ± 2.02^c^
TPUFAs	28.99 ± 1.24^bc^	29.94 ± 1.09^c^	29.71 ± 1.12^c^	28.56 ± 1.13^b^	28.18 ± 1.16^ab^	27.37 ± 1.17^a^
n-6 PUFAs	15.16 ± 0.57^bc^	15.33 ± 0.48^c^	15.27 ± 0.48^c^	14.74 ± 0.67^ab^	14.60 ± 0.66^a^	14.54 ± 0.58^a^
n-3 PUFAs	13.83 ± 0.76^bc^	14.67 ± 0.78^d^	14.44 ± 0.79^cd^	13.82 ± 0.5^b^	13.58 ± 0.62^b^	12.83 ± 0.74^a^
n-6/n-3 ratio	1.10 ± 0.04^bc^	1.04 ± 0.05^a^	1.06 ± 0.05^ab^	1.06 ± 0.03^ab^	1.07 ± 0.04^ab^	1.13 ± 0.05^c^
S/M/P	1.49 : 0.93 : 1	1.47 : 0.86 : 1	1.44 : 0.90 : 1	1.51 : 0.97 : 1	1.52 : 1.00 : 1	1.55 : 1.08 : 1

Fatty acid compositions (% area) in rats' heart						
16:00	12.68 ± 1.03^bc^	12.18 ± 0.33^ab^	13.13 ± 0.71^c^	11.92 ± 0.88^a^	12.50 ± 0.54^b^	12.11 ± 0.53^ab^
9c16:1	0.29 ± 0.54	0.11 ± 0.05	0.13 ± 0.05	0.14 ± 0.08	0.15 ± 0.07	0.11 ± 0.04
18:00	22.41 ± 1.62^b^	23.28 ± 0.56^c^	20.90 ± 0.85^a^	23.29 ± 0.76^c^	22.47 ± 0.62^b^	22.93 ± 0.94^bc^
9t18:1	0.15 ± 0.02^b^	0.14 ± 0.01^b^	0.18 ± 0.02^c^	0.14 ± 0.02^b^	0.12 ± 0.01^a^	0.12 ± 0.01^a^
9c18:1 OA	6.22 ± 2.34^bc^	5.02 ± 0.75^a^	6.41 ± 1.30^c^	5.65 ± 1.34^ac^	5.27 ± 0.93^ab^	5.18 ± 0.86^ab^
11c18:1	2.21 ± 0.14^b^	2.18 ± 0.06^b^	1.99 ± 0.13^a^	2.27 ± 0.21^b^	2.52 ± 0.12	2.49 ± 0.14^c^
9c12c18:2 LA	20.37 ± 1.40^a^	20.82 ± 0.95^a^	25.48 ± 2.39^b^	21.27 ± 1.90^a^	21.47 ± 1.30^a^	20.96 ± 0.92^a^
20:00	0.17 ± 0.02^bc^	0.18 ± 0.02^cd^	0.20 ± 0.04^d^	0.20 ± 0.04^d^	0.16 ± 0.01^a^	0.14 ± 0.01^a^
5c20:1	0.09 ± 0.03^ab^	0.09 ± 0.01^ab^	0.10 ± 0.01^b^	0.09 ± 0.01^ab^	0.08 ± 0.01^a^	0.09 ± 0.01^a^
11c20:1	0.16 ± 0.06^a^	0.14 ± 0.03^a^	0.40 ± 0.10^b^	0.27 ± 0.07^b^	0.14 ± 0.03^a^	0.14 ± 0.06^a^
18:3n-3 ALA	0.18 ± 0.07	0.16 ± 0.05	0.14 ± 0.04	0.14 ± 0.02	0.16 ± 0.02	0.18 ± 0.06^a^
20:2n-6	0.20 ± 0.02^b^	0.19 ± 0.02^ab^	0.19 ± 0.03^ab^	0.19 ± 0.02^ab^	0.19 ± 0.02^ab^	0.18 ± 0.01^a^
22:00	0.13 ± 0.02^cd^	0.12 ± 0.02^bc^	0.15 ± 0.03^d^	0.14 ± 0.04^cd^	0.10 ± 0.02^ab^	0.09 ± 0.01^a^
20:3n-3	0.61 ± 0.10^a^	0.60 ± 0.07^a^	1.13 ± 0.18^b^	0.68 ± 0.10^a^	0.62 ± 0.09^a^	0.54 ± 0.06^a^
20:4n-6 ARA	18.01 ± 0.66^cd^	18.08 ± 0.92^d^	15.33 ± 0.98^a^	16.91 ± 0.48^ab^	17.27 ± 0.86^bc^	17.75 ± 0.75^bd^
20:5n-3 EPA	0.19 ± 0.03^a^	0.21 ± 0.03^a^	0.28 ± 0.05^b^	0.20 ± 0.03^a^	0.20 ± 0.02^a^	0.21 ± 0.03
22:4n-6	0.44 ± 0.07^b^	0.37 ± 0.05^a^	0.36 ± 0.06^a^	0.34 ± 0.05^a^	0.35 ± 0.03^a^	0.37 ± 0.05^a^
22:5n-6	0.34 ± 0.08^b^	0.32 ± 0.03^ab^	0.32 ± 0.03^ab^	0.29 ± 0.04^a^	0.30 ± 0.04^a^	0.29 ± 0.03^a^
22:5n-3 DPA	0.69 ± 0.60^a^	1.83 ± 0.27^ab^	2.03 ± 0.35^b^	2.04 ± 0.27^b^	2.03 ± 0.23^b^	2.03 ± 0.32^b^
22:6n-3 DHA	12.47 ± 1.05^ab^	13.03 ± 0.83^ab^	12.31 ± 1.06^ab^	12.18 ± 1.25^a^	13.25 ± 1.15^b^	13.11 ± 1.00^b^
TSFAs	35.80 ± 1.08^b^	36.12 ± 0.53^b^	34.74 ± 1.24^a^	35.88 ± 0.90^b^	35.62 ± 0.63^b^	35.66 ± 0.85^b^
TMUFAs	9.25 ± 2.81^b^	7.81 ± 0.81^a^	9.37 ± 1.35^b^	8.72 ± 1.47^ab^	8.40 ± 1.10^ab^	8.27 ± 0.91^ab^
TPUFAs	54.35 ± 2.09^a^	55.61 ± 0.53^b^	55.50 ± 1.31^b^	55.01 ± 1.42^ab^	55.53 ± 1.01^b^	55.65 ± 0.68^b^
n-6 PUFAs	39.76 ± 1.87^a^	40.39 ± 0.67^a^	42.80 ± 2.19^b^	40.45 ± 1.63^a^	39.88 ± 1.49^a^	40.10 ± 1.02^a^
n-3 PUFAs	14.52 ± 1.31^b^	15.23 ± 0.83^bc^	14.76 ± 1.59^a^	14.56 ± 1.27^b^	15.6 ± 1.28^c^	15.53 ± 0.84^bc^
n-6/n-3 ratio	2.75 ± 0.31^a^	2.66 ± 0.19^a^	2.89 ± 0.49^b^	2.80 ± 0.32^a^	2.57 ± 0.32^a^	2.59 ± 0.19^a^
S/M/P	1 : 0.26 : 1.52	1 : 0.22 : 1.54	1 : 0.27 : 1.60	1 : 0.24 : 1.53	1 : 0.24 : 1.56	1 : 0.23 : 0.56

Values presented as means (*n* = 12) ± SD. Mean values within each row followed by different letters (a, b, c, etc.) are significantly (*P* < 0.05) different. OA, oleic acid; LA, linoleic acid; ALA, alpha-linolenic acid; EPA, eicosapntemacnioc acid; ARA, arachidonic acid; DPA, docosapentaenoic acid; DHA, docosahexenoic acid; TSFAs, total saturated fatty acids, the sum of (C16:0 + C18:0 + C20:0 + C22:0 + ⋯); TMUFAs, total monounsaturated fatty acids, the sum of (C16:1 + C18:1 + C20:1 + ⋯); TPUFAs, total polyunsaturated fatty acids, the sum of (C18:2n-6 + C18:3n-3 + C20:2n-6 + C20:3n-6 + C20:4n-6 + C20:5n-3 + C22:4n-6 + C22:5n-6 + C22:5n-3 + 22:6n-3 + ⋯); S/M/P: TSFAs/TMUFAs/TPUFAs. Different dietary treatments for groups: EG1 (0.8 mL blended oil, 9% palm oil, cholesterol of 0.04 g/kg total diet, and n-6/n-3 9 : 1); EG2 (0.8 mL blended oil, 9% palm oil, cholesterol of 0.057 g/kg total diet, and n-6/n-3 9 : 1); EG3 (0.8 mL blended oil, 8.6% palm oil, cholesterol of 0.04 g/kg total diet, and n-6/n-3 5 : 1); EG4 (0.8 mL blended oil, 8.6% palm oil, cholesterol of 0.057 g/kg total diet, and n-6/n-3 5 : 1); CG1 (0.6 mL blended oil, 6.1% palm oil, cholesterol of 0.04 g/kg total diet, and n-6/n-3 9 : 1); CG2 (0.6 mL blended oil, 6.1% palm oil, cholesterol of 0.057 g/kg total diet, and n-6/n-3 9 : 1). Different proportions of blended oil (g/100 g) for groups: EG1/EG2 (camellia oil 10 g, sunflower oil 74 g, and linseed oil 16 g); EG3/EG4 (camellia oil 18.6 g, sunflower oil 53.5 g, and linseed oil 27.9 g); CG1/CG2 (camellia oil 10.2 g, sunflower oil 73.5 g, and linseed oil 16.3 g).

## Abbreviations

BW: Body weight
PUFA: Polyunsaturated fatty acid
FAs: Fatty acids
TG: Triacylglycerols
TC: Total cholesterol
LDL-C: Low density lipoprotein cholesterol
HDL-C: High density lipoprotein cholesterol
VLDL-C: Very low density lipoprotein cholesterol
FAMEs: Fatty acid methyl esters
ARA: Arachidonic acid
EPA: Eicosapentaenoic acid
DHA: Docosahexaenoic acid
MUFA: Monounsaturated fatty acids
SFA: Saturated fatty acids
ALA: *α*-Linolenic acid.
